# Effectiveness of an exercise and nutrition intervention for older adults with mild cognitive impairment: an open-label double-arm clinical trial

**DOI:** 10.3389/fnagi.2025.1581400

**Published:** 2025-05-07

**Authors:** Moeko Noguchi-Shinohara, Kunihiko Yokoyama, Junji Komatsu, Kazumi Masuda, Mitsunobu Kouno, Mitsuhiro Yoshita, Kenjiro Ono

**Affiliations:** ^1^Department of Neurology, Kanazawa University Graduate School of Medical Sciences, Kanazawa, Japan; ^2^Department of Thyroidology, Public Central Hospital of Matto Ishikawa, Hakusan, Japan; ^3^Faculty of Human Sciences, Kanazawa University, Kanazawa, Japan; ^4^Faculty of Health Sciences, Kinjo University, Hakusan, Japan; ^5^Department of Neurology, National Hospital Organization, Hokuriku National Hospital, Nanto, Japan

**Keywords:** physical exercise, nutrition, intervention, dementia, mild cognitive impairment, cognitive decline

## Abstract

**Background:**

We examined the efficacy of physical exercise with nutritional lectures in preventing cognitive decline among older adults.

**Methods:**

This study included individuals aged ≥65 years who had no dementia. The intervention group underwent a physical exercise training program once a week and attended nutritional lectures once a month for 5 months. Baseline and 12-month cognitive assessments using the MCI Screen (MCIS) to measure memory performance were completed by individuals in the intervention (*n* = 76) and nonparticipant (*n* = 36) groups. The primary endpoint was changes in the memory performance index (MPI) scores of the MCIS.

**Results:**

The MPI score significantly increased by 1.8 in the intervention group and significantly decreased by 1.2 in the nonparticipant groups (*p* = 0.049).

**Discussion:**

Memory declines gradually overtime as a normal process of aging. Therefore, the decline of MPI score in the nonparticipant group is a natural change, however interestingly, the MPI scores improved in the intervention group, suggesting that the physical exercise with nutritional lectures may prevent memory function. Our results also suggest that through physical exercises and nutritional lectures, older adults might have developed exercise habits that increased their muscle weight which might have improved their cognitive function. A 5-month intervention of our physical exercise training program with nutritional lectures for older adults without dementia can improve their memory performance, especially individuals with 60s and 70s and with mild cognitive impairment.

## 1 Introduction

The increasing number of people with dementia worldwide has become a serious problem not only for people with dementia but also for their caregivers, the society (Alzheimer’s Association Report, 2023) and the costs (Wimo et al., 2023). Potentially modifiable lifestyle risk factors for dementia include diabetes, hypertension, obesity, and physical inactivity (Livingston et al., 2024). In particular, physical inactivity increases the risk of many adverse health conditions such as type 2 diabetes, depression (Lee et al., 2012), and dementia (Kulmala et al., 2014). Physical activity is thought to prevent and delay dementia (Erickson et al., 2011) by decreasing vascular damage, reducing dementia pathology, reducing stress and inflammation, and building cognitive and brain reserve (Kulmala et al., 2014). However, several older adults are physically inactive (Cabinet office, 2024).

In Hakusan City, which is in the southern region of Ishikawa Prefecture, Japan, approximately 112,800 residents and those ≥65 years old accounted for 28% of the population as of April 2022. We conducted an open-label, 12-month, double-arm study entitled “Effect of an exercise program to reduce risks of cognitive decline and physical frailty for older adults” (Noguchi-Shinohara et al., 2023) to examine the efficacy of an intervention program comprising physical exercise training and nutritional lectures to improve or maintain cognitive and physical functions. Furthermore, we investigated exploratory the relationship between body composition and cognition. The research hypothesis of this study are the exercise and nutrition intervention can prevent cognitive decline and that improve physical function in mild cognitive impairment among community-dwelling older adults.

## 2 Methods

### 2.1 Study design

This open-label, 12-month, double-arm trial was conducted in geriatric clinics and senior day care centers in Japan. The study protocol has been published elsewhere (Noguchi-Shinohara et al., 2023).

### 2.2 Participants

The only inclusion criterion was age ≥65 years. The exclusion criteria were as follows: (i) prohibition from exercise by a medical doctor, (ii) diagnosis of dementia, and (iii) nursing care level of the long-term care insurance (LTCI). In Japan, LTCI is a mandatory social insurance program that assists older adults with disability in their activities of daily living (Tamiya et al., 2011). Individuals who met the eligibility criteria were assigned to the intervention and nonparticipant groups if they chose to participate or not participate, respectively, in the intervention. Individuals in the nonparticipant group were asked to participate in cognitive check-ups at baseline and 12 months after.

This study was conducted in accordance with the guidelines of the Declaration of Helsinki, and all participants provided written informed consent for participation from Hakusan-city. The retrospective observational study to analyze data from the Hakusan-City project was approved by the Medical Ethics Review Board of Kanazawa University (Approval Number 114156-1). The trial was registered, and the study protocol was uploaded to the Japan Registry of Clinical Trials with the identifier jRCT 1,040,220,140. The protocol used the relevant standard protocol items for clinical trials according to the CONSORT statement (Noguchi-Shinohara et al., 2023).

### 2.3 Interventions

For 5 months, the intervention group underwent a physical exercise training program once a week and attended nutritional lectures once a month (Noguchi-Shinohara et al., 2023). The exercise program was individualized and comprised 15 min of multitask exercise, 20 min of resistance exercise, and 30 min of aerobic exercise for a total of 65 min per session. Resistance exercises included six main muscle groups, such as the quadriceps femoris, hamstring, latissimus dorsi and arm muscles, and abdominal and back muscles. Aerobic exercises included ergometer cycling or running on a treadmill (Noguchi-Shinohara et al., 2023). The nutritionist in this study provided nutritional lectures on low-salt diets, diet to prevent diabetes and dyslipidemia, and protein intake to prevent frailty for all participants in the intervention group (Noguchi-Shinohara et al., 2023).

### 2.4 Outcomes

The primary outcome was the change in the Memory Performance Index (MPI) scores of the MCI Screen (MCIS) from baseline to 12 months (Noguchi-Shinohara et al., 2023; Shankle et al., 2005; Trenkle et al., 2007). The MCIS is a 10-min, electronically scored, staff-administered test, derived from data mining and analysis of ADAS-Cog wordlist memory test (Shankle et al., 2009). The MCIS has been shown to both have high test/re-test and inter-rater reliability and is useful in tracking cognitive performance longitudinally, and it can distinguish among patterns of cognitive performance consistent with normal aging or MCI with 96 to 97% accuracy (Shankle et al., 2005; Trenkle et al., 2007; Shankle et al., 2009; Cho et al., 2008; Snyder et al., 2011; Rafii et al., 2011). The MPI quantifies the pattern of recalled and unrecalled words on a 0–100 scale, with lower scores indicating worsening cognition (Trenkle et al., 2007). In this study, we used a cutoff point of 50.2 to identify MCI (8). According to the baseline MPI score, we divided the participants into the MCI (MPI score < 50.2) and normal cognition (NC) (MPI score ≥50.2) groups. Both the intervention and nonparticipants group were cognitively assessed at baseline and on follow-up after 12 months.

The secondary outcomes included the following: (i) proportion of people who exercised regularly, (ii) incidence of MCI on the 12-month follow-up, (iii) changes in physical tests, (iv) changes in body composition, and (v) proportion of people who were frail (8). Frailty was assessed according to the self-reported Eleven-Check questionnaire, which has been validated as a frailty screening tool using a cutoff value of ≥5 points (Lyu et al., 2024).

### 2.5 Statistical analysis

Assuming an alpha error of 5% and a difference between the intervention and nonparticipant groups values of d = 0.5, the required sample size for an 80% power was 64 participants per group. Considering a 20% expected loss to follow-up, the final sample size was estimated to be 160, which was written in the study protocol (Noguchi-Shinohara et al., 2023). In this study, more than 80 individuals participated in both the intervention and nonparticipant groups, but the follow-up rate was low, especially in the nonparticipant group, with only 36 participants remaining after 12 months.

The primary efficacy analyses used generalized linear mixed effects for repeated measures to assess between-group differences in the MPI score changes from baseline to 12 months. Additionally, we conducted a sensitivity analysis including participants with nursing care levels of the long-term care insurance. The baseline characteristics of the intervention and nonparticipant groups were compared using t-test for the mean values of continuous variables and chi-square test for categorical variables. For secondary analyses, changes between baseline and 12-month parameters in the intervention group were analyzed using McNemar’s test. Spearman correlation was used to assess the association between the MPI score changes and body composition changes in the intervention group. A *p* value of <0.05 was considered statistically significant. Statistical analyses were performed using the Statistical Package for the Social Sciences software (version 28; SPSS Inc., Chicago, IL, United States).

## 3 Results

### 3.1 Study population and baseline characteristics

**Figure 1:**
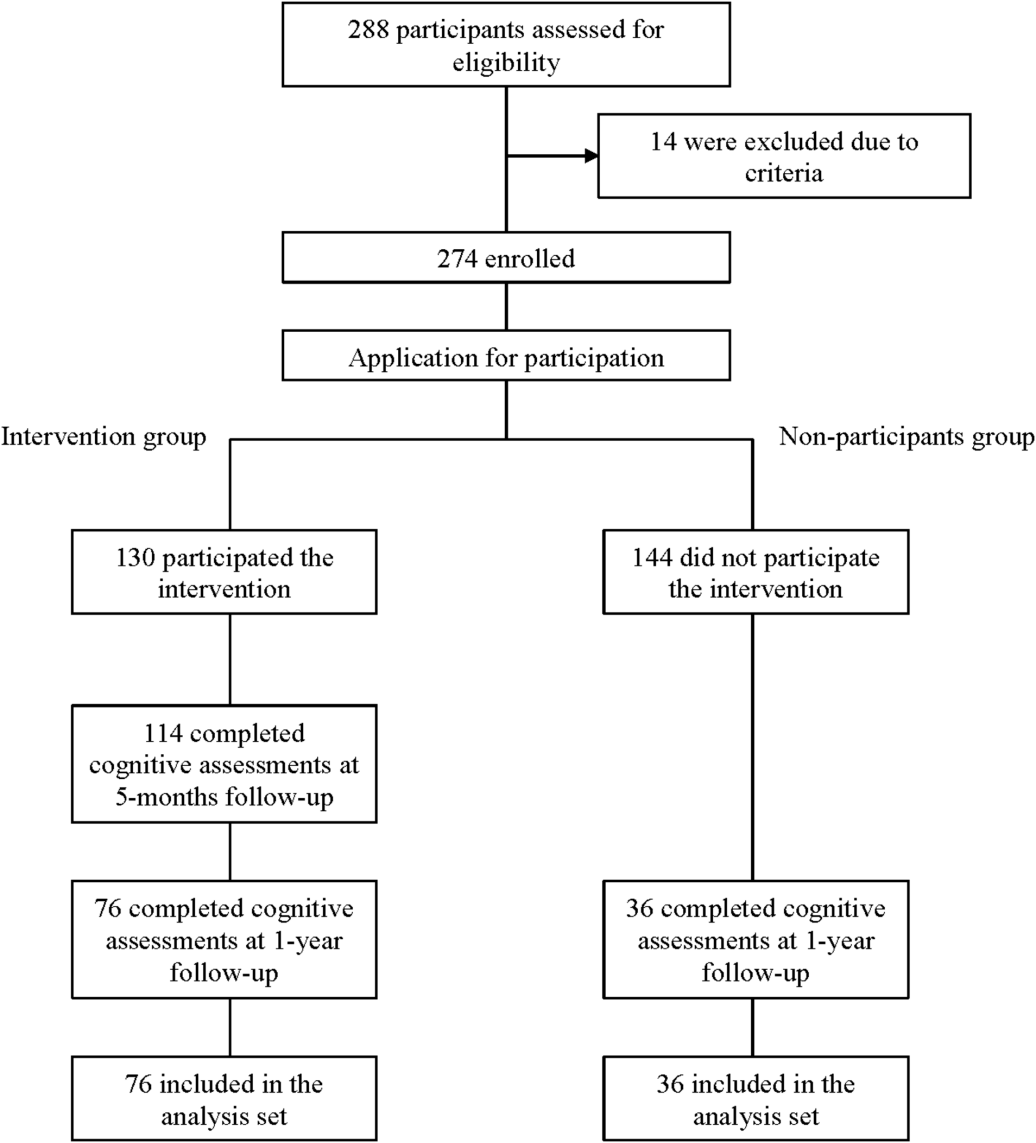
Participants flow.

Between September 2022 and October 2022, 288 individuals were screened, and 274 were enrolled in this study (Figure 1). The assessments at 12 months were completed by 76 (58.4%) participants in the intervention group (*n* = 130) and by 36 (25.0%) individuals in the nonparticipant group (*n* = 144). We compared between 36 individuals who underwent the follow-up assessment (follow-up assessment group, i.e., nonparticipants group) and 108 individuals who did not undergo the follow-up assessment at 12 months (non-follow-up assessment group). There were no significant differences in age and sex, however the MPI score at baseline was significantly lower and the proportion of individuals with MCI was significantly higher in the non-follow-up assessment group (Supplementary Table 1). The baseline characteristics of the intervention and nonparticipant groups are presented in Table 1. The intervention and nonparticipant groups were similar in terms of the proportion of women (86.8% vs. 75.0%, respectively; *p* = 0.176). Those aged ≥80 years were no statistically significant differences (46.1% vs. 22.2%, respectively; *p* = 0.072). At baseline, the MPI score was significantly lower (*p* = 0.002) and the proportion of individuals with MCI was significantly higher (*p* = 0.029) in the intervention group than in the nonparticipant group. In addition, use of LTCI at the level of needed support was significantly more frequent in the intervention group than in the nonparticipant group (25.0% vs. 0%, *p* < 0.001).

**Table 1:** Baseline characteristics of the study participants.

Variables	Non-participants group	Intervention group	*p*
*n*	36	76	
No. of women, *n* (%)	27 (75.0)	66 (86.8)	0.176
Age			0.072
65 ~ 69 years old, *n* (%)	2 (5.6)	7 (9.2)	
70 ~ 79 years old, *n* (%)	26 (72.2)	34 (44.7)	
80 years old and over, *n* (%)	8 (22.2)	35 (46.1)	
Regular exercise	ND	18 (23.6)	
Comorbidities			
Diabetes	ND	12 (15.7)	
Hypertension	ND	35 (46.0)	
Dyslipidemia	ND	19 (25.0)	
MPI score, mean ± SD	57.1 ± 8.2	51.2 ± 12.7	0.002
MCI, *n* (%)	6 (16.7)	29 (38.1)	0.029
Use of LTCI, *n* (%)	0	19 (25.0)	< 0.001

LTCI, long-term care insurance; MCI, mild cognitive impairment; MPI, memory performance index; ND, no data.

### 3.2 Intervention effects on cognition

**Figure 2:**
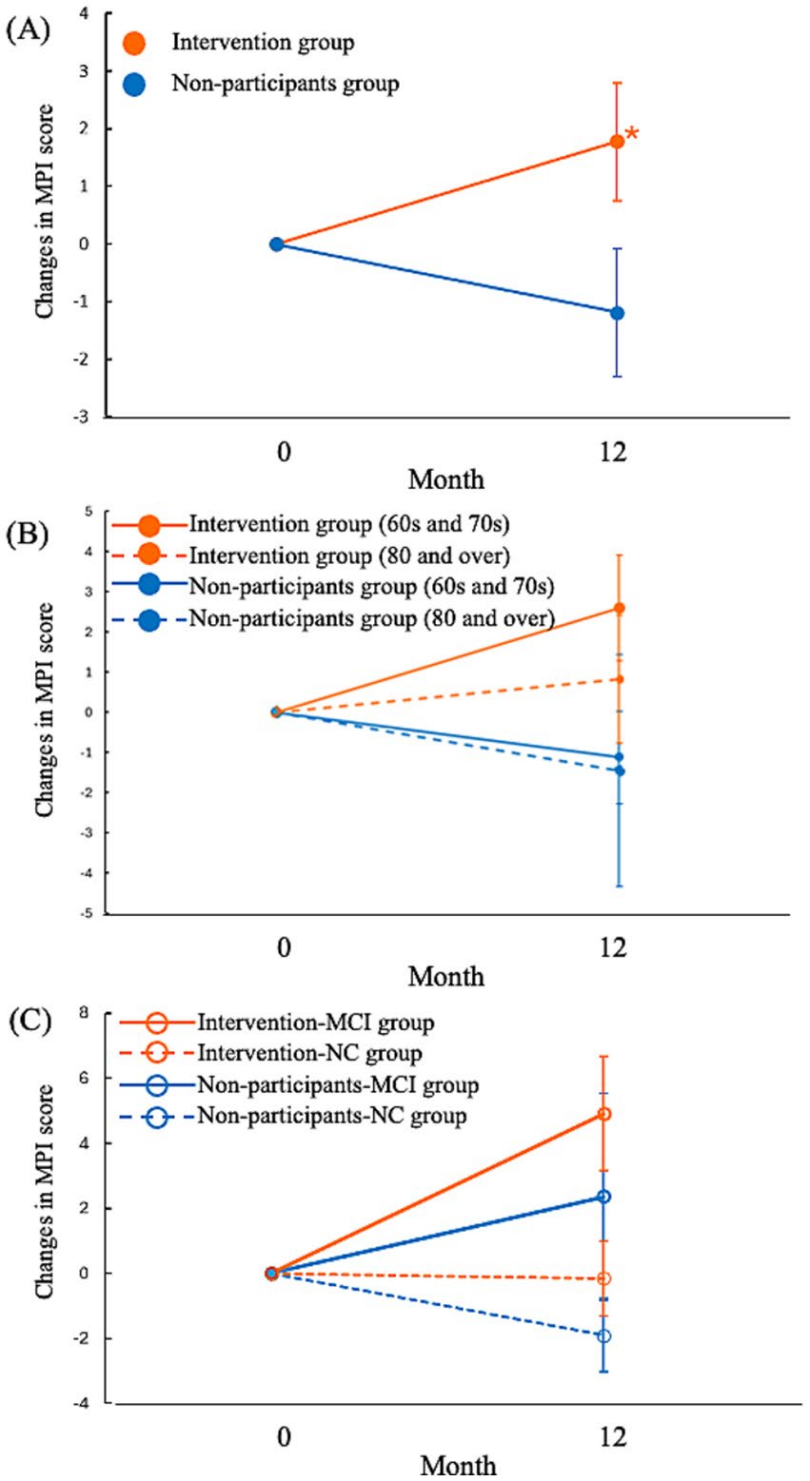
Change in the MPI from baseline to 12 months after intervention. **(A)** The estimated mean change in the MPI from baseline to 12 months is shown (with higher scores indicating better performance). Error bars represent standard errors. *The mean difference in the MPI score changes between the intervention and nonparticipant groups was significant (*p* < 0.05). **(B)** The estimated mean change in the MPI from baseline to 12 months in the age groups 60s and 70s and ≥80 years. Error bars represent the standard errors. **(C)** The estimated mean change in the MPI from baseline to 12 months in the MCI and NC groups. Error bars represent the standard errors. MCI, mild cognitive impairment; MPI, memory performance index; NC, normal cognition.

The intervention had a significant beneficial effect on the MPI score (Figure 2A and Supplementary Table 2A). The estimated mean (standard error) change in the MPI score at 12 months was significantly different between the intervention and nonparticipant groups [1.8 (1.0) vs. −1.2 (1.1), respectively; mean difference 4.42, 95% CI 0.02–8.83; *p* = 0.049]. In the stratified analysis according to age, the MPI scores improved to a greater degree among individuals in their 60s and 70s than in those ≥80 years old in the intervention group but declined to a greater degree among individuals ≥80 years old than in those in their 60s and 70s in the nonparticipant group (Figure 2B and Supplementary Table 2B). In the stratified analysis according to preintervention cognition, the intervention–MCI group showed the greatest improvement in MPI, whereas the nonparticipant–NC group showed the greatest deterioration in MPI (Figure 2C and Supplementary Table 2C). Sensitivity analysis including participants with nursing care levels of the LTCI also revealed that the intervention had a significant beneficial effect on the MPI score (Supplementary Tables 3A, B).

At 12 months, the proportion of individuals with MCI decreased in the intervention group from 38.1 to 34.2%, whereas it increased in the nonparticipant group from 16.6 to 22.2%. During the 12-month follow-up, the number of people with MCI increased from 6 to 8 in the nonparticipant group, whereas it decreased from 29 to 26 in the intervention group (Supplementary Table 4).

### 3.3 Intervention effects on the secondary endpoints

**Figure 3:**
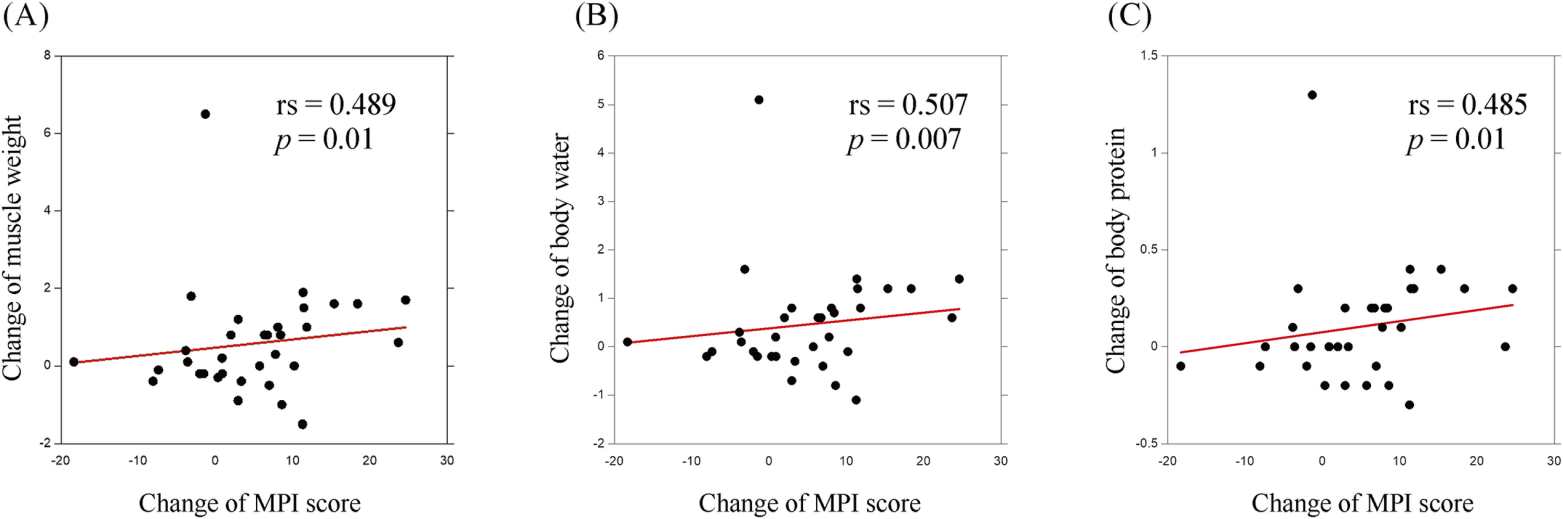
Association between the MPI score change and body composition change. The associations of the change in MPI score with the changes in **(A)** muscle weight, **(B)** body water, and **(C)** body protein are shown. MPI, memory performance index.

For the other secondary endpoints, the proportion of people who exercised regularly significantly increased in the intervention group from 23.6 to 42.1% (*p* = 0.004) (Supplementary Table 4), suggesting that the exercise program was effective in establishing exercise habits. Among the physical functions, the mean time to stand on one foot with open eyes significantly increased in the intervention group from 38.2 s to 49.7 s (*p* = 0.01) (Supplementary Table 4). In terms of body composition, muscle weight and body water, body protein, and body mineral significantly increased, whereas body weight and body fat significantly decreased in the intervention group (Supplementary Table 4). Interestingly, among participants in the intervention group who had MCI at baseline, changes in the MPI scores at 12 months significantly correlated with the changes in muscle weight (rs = 0.489, *p* = 0.01); body water (rs = 0.507, *p* = 0.007); and body protein (rs = 0.485, *p* = 0.01) (Figure 3). In the intervention group, there were no significant changes from baseline to 12 months in grip strength, sit and reach test, and frailty status.

### 3.4 Adverse events

One participant in the intervention group sustained a fall. No serious adverse events were reported.

## 4 Discussion

This study showed that a 5-month intervention of physical exercise and nutritional lectures increased the MPI scores of community-dwelling older adults who had no dementia which might mean an exercise and nutrition intervention prevents cognitive decline. The stratified analysis revealed that greater improvement of MPI scores in intervention-MCI group than in the NC group, and in the intervention-60s and 70s than in the 80s age group. Moreover, among those with MCI at baseline, changes in the cognition and body composition measures from baseline to 12 months after intervention were found to have significant correlations. In other words, the intervention in this study increased muscle weight, body water, and body protein and improved MPI scores. It is possible that as muscle weight increases, glucose intolerance and blood pressure improve (Usui, 2023), and these confounding factors might contribute to prevent cognitive decline. However, glucose intolerance and blood pressure were not measured in this study, further investigations including them are required. A previous study has shown significant relationships between sarcopenia and cognitive impairment (Cabett Cipolli et al., 2019). The mechanism of this association remains unclear, although several explanations are possible. Physical inactivity caused by sarcopenia may result in reduced expression and low serum levels of brain-derived neurotrophic factor (BDNF) and insulin growth factor (IGF-1), which were reported to be associated with physical inactivity (Kang and Schuman, 1995; Angulo et al., 2020). However, physical exercise could affect BDNF and IGF-1 expressions and lead to cognitive improvement.

In the intervention group, the number of people who developed exercise habits increased, and physical function was maintained or improved. Surprisingly, most body composition indices improved in a desired direction. In addition, adverse events, such as falls, were rare. Unfortunately, the proportion of people with high scores on the frailty index did not significantly change from baseline to 12 months in the intervention group. Although this result may seem contradictory to an increase in muscle weight and the improvements of MPI scores, the frailty index at least did not worsen and may even improve over the longer term. In our study, the intervention period was 5 months, and the primary outcome was assessed 12 months after the baseline, meaning there was a 7-month gap. During the 7-month gap period, no other interventions were undertaken. People in the intervention group were encouraged to continue their exercise and healthy meals after the physical exercise training program and nutritional lectures had ended in 5 months.

Multidomain intervention studies have demonstrated mixed outcomes; some showed improved cognitive function (Ngandu et al., 2015; Oki et al., 2024), whereas others did not show significant efficacy in preventing cognitive decline (van Charante et al., 2016; Andrieu et al., 2017; Bischoff-Ferrari et al., 2020; Sakurai et al., 2024). However, in some studies, subgroup analyses showed the efficacy of multidomain intervention among groups with good adherence to the intervention or high risk of cognitive decline, such as those who had positive amyloid PET scan, carried the apolipoprotein E ε4 allele, and had a high CAIDE dementia risk score (Andrieu et al., 2017; Sakurai et al., 2024). Similarly, our results showed that the intervention was more effective in the MCI group who was at a high risk of cognitive decline (Andrieu et al., 2017; Sakurai et al., 2024). In terms of age, we found that the intervention improved the MPI scores among individuals in their 60s and 70s but not among those ≥80 years old. Consistently, a previous study showed that multidomain intervention improved cognitive function in young older people (65–74 years old) but not in old older people (75–89 years old) (Sakurai et al., 2024).

Our study found that the intervention prevented memory performance decline. In the Finnish Geriatric Intervention Study to Prevent Cognitive Impairment and Disability, the executive function/processing speed cognitive domain was improved by a multidomain intervention (Ngandu et al., 2015). Likewise, the Japan-Multimodal Intervention Trial for Prevention of Dementia PRIME Tamba showed improvement of the memory domain, in addition to the executive function/processing speed domain (Oki et al., 2024). In this study, we did not examine the executive function/processing speed of the participants. Our intervention of physical exercise and nutritional lectures were group-based intervention can also improve social isolation which is potentially modifiable lifestyle risk factor (Livingston et al., 2024). Placebo effects play a role independently of actual exercise in the health benefits of exercise (Crum et al., 2007). These factors, such as socialization and placebo effect may have contributed to preventing cognitive decline in this study.

The limitations of this study were as follows: (i) nonrandomized control design, and we observed significantly worse cognitive function and higher proportion of people with MCI in the intervention group than in the nonparticipants group in baseline characteristics. (ii) various cognitive tests other than episodic memory were not examined, (iii) some baseline characteristics data of the non-participants group were missing such as exercise habits, comorbidities (diabetes, hypertension and dyslipidemia), and body weight, (iv) short follow-up period of 12 months, and (v) low follow-up rates (58.4% in the intervention group and 25.0% in the nonparticipant group). Therefore, response bias may have influenced the results of this study. In the future, a randomized controlled trial with longer study periods, various cognitive tests, and larger number of participants are required to prove the efficacy of this intervention program. The strengths of this study were as follows: (i) inclusion of community-dwelling older adults with a wide age range, (ii) demonstration of significant memory improvement after only 5 months of the intervention, and (iii) demonstration of the safety of the intervention program, based on only one adverse event, which was a fall during physical fitness test.

In conclusion, a 5-month intervention of physical exercise and nutrition lectures was found to be effective in preventing the cognition of older adults, especially people in their 60s and 70s and including those with MCI, after 12 months.

## Data Availability

The data are not publicly available due to privacy or ethical restrictions. However, the data are available on reasonable request and with the permission of the corresponding author.

## References

[ref1] Alzheimer’s Association Report (2023). 2023 Alzheimer’s disease facts and figures. Alzheimers Dement. 19, 1598–1695. doi: 10.1002/alz.13016, PMID: 36918389

[ref2] Andrieu S. Guyonnet S. Coley N. Cantet C. Bonnefoy M. Bordes S.. (2017). Effect of long-term omega 3 polyunsaturated fatty acid supplementation with or without multidomain intervention on cognitive function in elderly adults with memory complaints (MAPT): a randomised, placebo-controlled trial. Lancet Neurol. 16, 377–389. doi: 10.1016/S1474-4422(17)30040-6, PMID: 28359749

[ref3] Angulo J. El Assar M. Álvarez-Bustos A. Rodríguez-Mañas L. (2020). Physical activity and exercise: strategies to manage frailty. Redox Biol. 35:101513. doi: 10.1016/j.redox.2020.101513, PMID: 32234291 PMC7284931

[ref4] Bischoff-Ferrari H. A. Vellas B. Rizzoli R. Kressig R. W. da Silva J. A. P. Blauth M.. (2020). Effect of vitamin D supplementation, Omega-3 fatty acid supplementation, or a strength-training exercise program on clinical outcomes in older adults: the DO-HEALTH randomized clinical trial. JAMA 324, 1855–1868. doi: 10.1001/jama.2020.16909, PMID: 33170239 PMC7656284

[ref5] Cabett CipolliG.Sanches YassudaM.AprahamianI. (2019). Sarcopenia is associated with cognitive impairment in older adults: a systematic review and Meta-analysis. J. Nutr. Health Aging 23, 525–531. doi: 10.1007/s12603-019-1188-8, PMID: 31233073

[ref6] Cabinet office, Annual Report on the Ageing Society 2024 (2024). Available at: https://www8.cao.go.jp/kourei/whitepaper/w-2024/html/zenbun/s1_2_2.html (in Japanese).

[ref7] Cho A. Sugimura M. Nakano S. Yamada T. (2008). The Japanese MCI screen for early detection of Alzheimer’s disease and related disorders. Am. J. Alzheimers Dis. Other Dement. 23, 162–166. doi: 10.1177/1533317507312624, PMID: 18223126 PMC10846169

[ref900] Crum A. J. Langer E. J. (2007). Mind-set matters: Exercise and the placebo effect. Psychological Science 18, 165–171., PMID: 17425538 10.1111/j.1467-9280.2007.01867.x

[ref8] Erickson K. I. Voss M. W. Prakash R. S. Basak C. Szabo A. Chaddock L.. (2011). Exercise training increases size of Hippocampus and improves memory. Proc. Natl. Acad. Sci. USA 108, 3017–3022. doi: 10.1073/pnas.1015950108, PMID: 21282661 PMC3041121

[ref9] Kang H. Schuman E. M. (1995). Long-lasting Neurotrophin-induced enhancement of synaptic transmission in the adult Hippocampus. Science 267, 1658–1662. doi: 10.1126/science.7886457, PMID: 7886457

[ref10] Kulmala J. Solomon A. Kåreholt I. Ngandu T. Rantanen T. Laatikainen T.. (2014). Association between mid- to late life physical fitness and dementia: evidence from the CAIDE study. J. Intern. Med. 276, 296–307. doi: 10.1111/joim.12202, PMID: 24444031

[ref11] Lee I. M. Shiroma E. J. Lobelo F. Puska P. Blair S. N. Katzmarzyk P. T.. (2012). Effect of physical inactivity on major non-communicable diseases worldwide: an analysis of burden of disease and life expectancy. Lancet (London, England) 380, 219–229. doi: 10.1016/S0140-6736(12)61031-9, PMID: 22818936 PMC3645500

[ref12] Livingston G. Huntley J. Liu K. Y. Costafreda S. G. Selbæk G. Alladi S.. (2024). Dementia prevention, intervention, and care: 2024 report of the lancet standing commission. Lancet (London, England) 404, 572–628. doi: 10.1016/S0140-6736(24)01296-0, PMID: 39096926

[ref13] Lyu W. Tanaka T. Son B.-K. Yoshizawa Y. Akishita M. Iijima K. (2024). Validity of a simple self-reported questionnaire “eleven-check” for screening of frailty in Japanese community-dwelling older adults: Kashiwa cohort study. Arch. Gerontol. Geriatr. 117:105257. doi: 10.1016/j.archger.2023.105257 37952422

[ref14] Ngandu T. Lehtisalo J. Solomon A. Levälahti E. Ahtiluoto S. Antikainen R.. (2015). A 2 year multidomain intervention of diet, exercise, cognitive training, and vascular risk monitoring versus control to prevent cognitive decline in at-risk elderly people (FINGER): a randomised controlled trial. Lancet 385, 2255–2263. doi: 10.1016/S0140-6736(15)60461-5, PMID: 25771249

[ref15] Noguchi-Shinohara M. Yokoyama K. Komatsu J. Masuda K. Kouno M. Yoshita M.. (2023). Exercise program to reduce the risk of cognitive decline and physical frailty in older adults: study protocol for an open label double-arm clinical trial. Front. Aging Neurosci. 15:1162765. doi: 10.3389/fnagi.2023.1162765, PMID: 37273649 PMC10235445

[ref16] Oki Y. Osaki T. Kumagai R. Murata S. Encho H. Ono R.. (2024). An 18-month multimodal intervention trial for preventing dementia: J-MINT PRIME Tamba. Alzheimers Dement. 20, 6972–6983. doi: 10.1002/alz.14170, PMID: 39229900 PMC11485327

[ref17] Rafii M. S. Taylor C. Coutinho A. Kim K. Galasko D. (2011). Comparison of the memory performance index with standard neuropsychological measures of cognition. Am. J. Alzheimers Dis. Other Dement. 26, 235–239. doi: 10.1177/1533317511402316, PMID: 21406427 PMC3568924

[ref18] Sakurai T. Sugimoto T. Akatsu H. Doi T. Fujiwara Y. Hirakawa A.. (2024). Japan-multimodal intervention trial for the prevention of dementia: a randomized controlled trial. Alzheimers Dement. 20, 3918–3930. doi: 10.1002/alz.13838, PMID: 38646854 PMC11180858

[ref19] Shankle W. R. Kimball Romney A. Hara J. Fortier D. Dick M. B. Chen J. M.. (2005). Methods to improve the detection of mild cognitive impairment. Proc. Natl. Acad. Sci. USA 102, 4919–4924. doi: 10.1073/pnas.0501157102, PMID: 15781874 PMC555034

[ref20] Shankle W. R. Mangrola T. Chan T. Hara J. (2009). Development and validation of the memory performance index: reducing measurement error in recall tests. Alzheimers Dement. 5, 295–306. doi: 10.1016/j.jalz.2008.11.001 19560100

[ref21] Snyder P. J. Jackson C. E. Petersen R. C. Khachaturian A. S. Kaye J. Albert M. S.. (2011). Assessment of cognition in mild cognitive impairment: a comparative study. Alzheimers Dement. 7, 338–355. doi: 10.1016/j.jalz.2011.03.009, PMID: 21575877 PMC4042858

[ref22] Tamiya N. Noguchi H. Nishi A. Reich M. R. Ikegami N. Hashimoto H.. (2011). Population ageing and wellbeing: lessons from Japan’s long-term care insurance policy. Lancet (London, England) 378, 1183–1192. doi: 10.1016/S0140-6736(11)61176-8, PMID: 21885099

[ref23] Trenkle D. L. Shankle W. R. Azen S. P. (2007). Detecting cognitive impairment in primary care: performance assessment of three screening instruments. J. Alzheimer’s Dis. 11, 323–335. doi: 10.3233/jad-2007-11309, PMID: 17851183

[ref24] Usui I. (2023). Common metabolic features of hypertension and type 2 diabetes. Hypertens. Res. 46, 1227–1233. doi: 10.1038/s41440-023-01233-x, PMID: 36869145

[ref25] van Charante M. Eric P. Richard E. Eurelings L. S. van Dalen J.-W. Ligthart S. A.. (2016). Effectiveness of a 6-year multidomain vascular care intervention to prevent dementia (preDIVA): a cluster-randomised controlled trial. Lancet (London, England) 388, 797–805. doi: 10.1016/S0140-6736(16)30950-3, PMID: 27474376

[ref26] Wimo A. Seeher K. Cataldi R. Cyhlarova E. Dielemann J. L. Frisell O.. (2023). The worldwide costs of dementia in 2019. Alzheimers Dement. 19, 2865–2873. doi: 10.1002/alz.12901, PMID: 36617519 PMC10842637

