# Supplementary material for: Effectiveness of an exercise and nutrition intervention for older adults with mild cognitive impairment: an open-label double-arm clinical trial
Source: Front Aging Neurosci. 2025 May 7;17:1581400. doi: 10.3389/fnagi.2025.1581400 (PMC12092448; doi:10.3389/fnagi.2025.1581400)
Supplement: Supplementary file 1 [file Table_1.DOCX]

**Supplementary table 1. Comparison between follow-up assessment and non-follow-up assessment groups among nonparticipants of the intervention.**

|  | Follow-up assessment group | Non-follow-up assessment group |  |
| --- | --- | --- | --- |
|  | (Nonparticipants group) |  | *p* |
| n | 36 | 108 |  |
| No. of women, n (%) | 27 (75.0) | 93 (86.1) | 0.128 |
| Age |  |  | 0.188 |
| 65~69 years old, n (%) | 2 (5.6) | 7 (6.5) |  |
| 70~79 years old, n (%) | 26 (72.2) | 61 (56.5) |  |
| 80 years old and over, n (%) | 8 (22.2) | 40 (37.0) |  |
| MPI score, mean ± SD | 57.1 ± 8.2 | 51.8 ± 12.1 | 0.004 |
| MCI, n (%) | 6 (16.7) | 42 (38.9) | 0.015 |
| Use of LTCI, n (%) | 0 | 0 | - |

Abbreviations: LTCI, long-term care insurance; MCI, mild cognitive impairment; MPI, memory performance index.
