# Supplementary material for: Effectiveness of an exercise and nutrition intervention for older adults with mild cognitive impairment: an open-label double-arm clinical trial
Source: Front Aging Neurosci. 2025 May 7;17:1581400. doi: 10.3389/fnagi.2025.1581400 (PMC12092448; doi:10.3389/fnagi.2025.1581400)
Supplement: Supplementary file 2 [file Table_2.DOCX]

**Supplementary table 2A. Change in MPI score during baseline and 12-month follow-up.**

|  | Non-participants group | Intervention group | *p* | η^2^ |
| --- | --- | --- | --- | --- |
| MPI score |  |  |  |  |
| Baseline | 57.1 ± 8.2 | 51.2 ± 12.7 |  |  |
| 1-year follow-up | 55.9 ± 8.0 | 53.0 ± 13.4 | 0.049 | 0.035 |

**Supplementary table 2B. Change in MPI score during baseline and 12-month follow-up according to age group.**

|  | Non-participants group | | Intervention group | | |
| --- | --- | --- | --- | --- | --- |
|  | 60s and 70s  n = 28 | 80 and over  n = 8 | | 60s and 70s  n = 41 | 80 and over  n = 35 |
| MPI score |  |  | |  |  |
| Baseline | 59.2 ± 6.1 | 49.8 ± 10.6 | | 57.6 ± 8.9 | 43.7 ± 12.3 |
| 1-year follow-up | 58.0 ± 5.7 | 48.4 ± 10.5 | | 60.2 ± 8.7 | 44.5 ± 13.1 |

**Supplementary table 2C. Change in MPI score during baseline and 12-month follow-up according to pre-intervention cognition.**

|  | Non-participants group | | Intervention group | | |
| --- | --- | --- | --- | --- | --- |
|  | NC  n = 30 | MCI  n = 6 | | NC  n = 47 | MCI  n = 29 |
| MPI score |  |  | |  |  |
| Baseline | 59.8 ± 5.0 | 43.6 ± 7.7 | | 59.4 ± 5.7 | 37.9 ± 9.0 |
| 1-year follow-up | 57.9 ± 5.9 | 45.9 ± 10.0 | | 59.2 ± 9.3 | 42.8 ± 13.0 |

Abbreviations: MCI, mild cognitive impairment; MPI, memory performance index; NC, normal cognition.
