# Supplementary material for: Effectiveness of an exercise and nutrition intervention for older adults with mild cognitive impairment: an open-label double-arm clinical trial
Source: Front Aging Neurosci. 2025 May 7;17:1581400. doi: 10.3389/fnagi.2025.1581400 (PMC12092448; doi:10.3389/fnagi.2025.1581400)
Supplement: Supplementary file 3 [file Table_3.DOCX]

**Supplementary table 3A. Baseline characteristics of the study participants including nursing care level of the LTCI.**

|  | Non-participants group | Intervention group | *p* |
| --- | --- | --- | --- |
| n | 36 | 82 |  |
| No. of women, n (%) | 27 (75.0) | 71 (86.6) | 0.181 |
| Age |  |  | 0.035 |
| 65~69 years old, n (%) | 2 (5.6) | 7 (8.5) |  |
| 70~79 years old, n (%) | 26 (72.2) | 35 (42.7) |  |
| 80 years old and over, n (%) | 8 (22.2) | 40 (48.8) |  |
| Regular exercise | ND | 19 (23.1) |  |
| Comobidities |  |  |  |
| Diabetes | ND | 12 (14.6) |  |
| Hypertension | ND | 37 (45.1) |  |
| Dyslipidemia | ND | 19 (23.1) |  |
|  |  |  |  |
| MPI score, mean ± SD | 57.1 ± 8.2 | 49.3 ± 14.2 | < 0.001 |
| MCI, n (%) | 6 (16.7) | 35 (42.6) | 0.007 |
| Use of LTCI, n (%) | 0 | 25 (30.4) | < 0.001 |

Abbreviations: LTCI, long-term care insurance; MCI, mild cognitive impairment; MPI, memory performance index.

**Supplementary table 3B. Sensitivity analysis of the primary cognitive endpoint from baseline and 12-month follow ups.**

|  | Non-participants group | Intervention group | *p* | η^2^ |
| --- | --- | --- | --- | --- |
| MPI score |  |  |  |  |
| Baseline | 57.1 ± 8.2 | 49.3 ± 14.2 |  |  |
| 1-year follow-up | 55.9 ± 8.0 | 51.1 ± 14.9 | 0.012 | 0.053 |
