# Supplementary material for: Effectiveness of an exercise and nutrition intervention for older adults with mild cognitive impairment: an open-label double-arm clinical trial
Source: Front Aging Neurosci. 2025 May 7;17:1581400. doi: 10.3389/fnagi.2025.1581400 (PMC12092448; doi:10.3389/fnagi.2025.1581400)
Supplement: Supplementary file 4 [file Table_4.DOCX]

**Supplementary table 4. Secondary endpoints measurements.**

Abbreviation: ND, no data
